# Amphetamine-Induced Sensitization Has Little Effect on Multiple Learning Paradigms and Fails to Rescue Mice with a Striatal Learning Defect

**DOI:** 10.1371/journal.pone.0059964

**Published:** 2013-04-15

**Authors:** Kiara C. Eldred, Richard D. Palmiter

**Affiliations:** 1 Department of Biochemistry at the University of Washington, Seattle, Washington, United States of America; 2 Howard Hughes Medical Institute, University of Washington, Seattle, Washington, United States of America; Louisiana State University Health Sciences Center, United States of America

## Abstract

Behavioral sensitization to psychostimulants such as amphetamine (AMPH) is associated with synaptic modifications that are thought to underlie learning and memory. Because AMPH enhances extracellular dopamine in the striatum where dopamine and glutamate signaling are essential for learning, one might expect that the molecular and morphological changes that occur in the striatum in response to AMPH, including changes in synaptic plasticity, would affect learning. To ascertain whether AMPH sensitization affects learning, we tested wild-type mice and mice lacking NMDA receptor signaling in striatal medium spiny neurons in several different learning tests (motor learning, Pavlovian association, U-maze escape test with strategy shifting) with or without prior sensitization to AMPH. Prior sensitization had minimal effect on learning in any of these paradigms in wild-type mice and failed to restore learning in mutant mice, despite the fact that the mutant mice became sensitized by the AMPH treatment. We conclude that the changes in synaptic plasticity and many other signaling events that occur in response to AMPH sensitization are dissociable from those involved in learning the tasks used in our experiments.

## Introduction

Psychostimulants and most other drugs of abuse elevate dopamine signaling, which is thought to amplify normal dopaminergic processes involved in motivation and learning. Repeated exposure to psychostimulants leads to changes in dopamine neurons within the ventral tegmental area (VTA) and their post-synaptic targets in the nucleus accumbens (NAc) as well as other brain regions. Many of these alterations last for weeks or months after the final exposure to the drug. Changes in glutamatergic signaling, intracellular signaling, transcription and chromatin structure have been extensively documented [1]–[6]. The synaptic changes that occur during prolonged exposure to psychostimulants are associated with the appearance of more dendritic spines on striatal medium spiny neurons (MSNs), which are thought to reflect enhanced excitatory input onto MSNs [7]–[9]. Changes in the AMPA to NMDA receptor ratio and subunit composition of AMPA receptors in both of these brain regions have been extensively analyzed and correlated with long-term potentiation (LTP) and long-term depression (LTD) of excitatory synapses following both acute and chronic exposure to drugs of abuse [2], [10]–[18]. The action of psychostimulants, especially AMPH, leads to greatly elevated extracellular dopamine at dopaminergic terminals and dendrites, and hence has the potential to induce synaptic changes broadly throughout the VTA and NAc, as well as other brain regions [1], [19]–[21]. In addition to the cellular and molecular changes outlined above, repeated exposure to psychostimulants gradually enhances behavioral response to the drug, which is usually measured as enhanced locomotion that persists for months after the final drug injection [22].

Dopamine-deficient mice are unmotivated, will not perform any goal-directed behaviors and fail to learn all but the simplest tasks [23]; however, restoration of dopamine signaling to the striatum of dopamine-deficient mice by gene therapy approaches is sufficient to restore the ability to learn most cognitive behaviors [24]. Dopamine signaling in the striatum is thought to enhance strong glutamatergic signals from the cortex, thalamus, amygdala, and hippocampus while attenuating weak signals, thereby highlighting relevant inputs and facilitating goal-directed behaviors. Glutamatergic signaling via NMDA receptors is also essential within MSNs for learning all tasks that have been examined [25], [26]. NMDA receptor signaling is essential to generate LTP in both dopamine neurons and MSNs [25]–[30]. Synaptic changes have also been detected in VTA dopamine neurons when performed at a critical stage in learning [31]–[32]. Taken together, these studies showing an association of synaptic plasticity mediated by NMDA receptors with learning in other brain regions suggests that NMDA receptor-dependent synaptic plasticity in dopamine neurons and/or MSNs underlies learning.

We tested the effect of prior AMPH sensitization on the ability of mice to learn several tasks. Because sensitization still occurs in mice without NMDA receptors in MSNs [33], we were also curious to learn whether the synaptic changes that occur during sensitization would restore the learning ability of mice lacking NMDA receptors in MSNs.

## Results

### Sensitization of wild-type mice to AMPH

**Figure 1:**
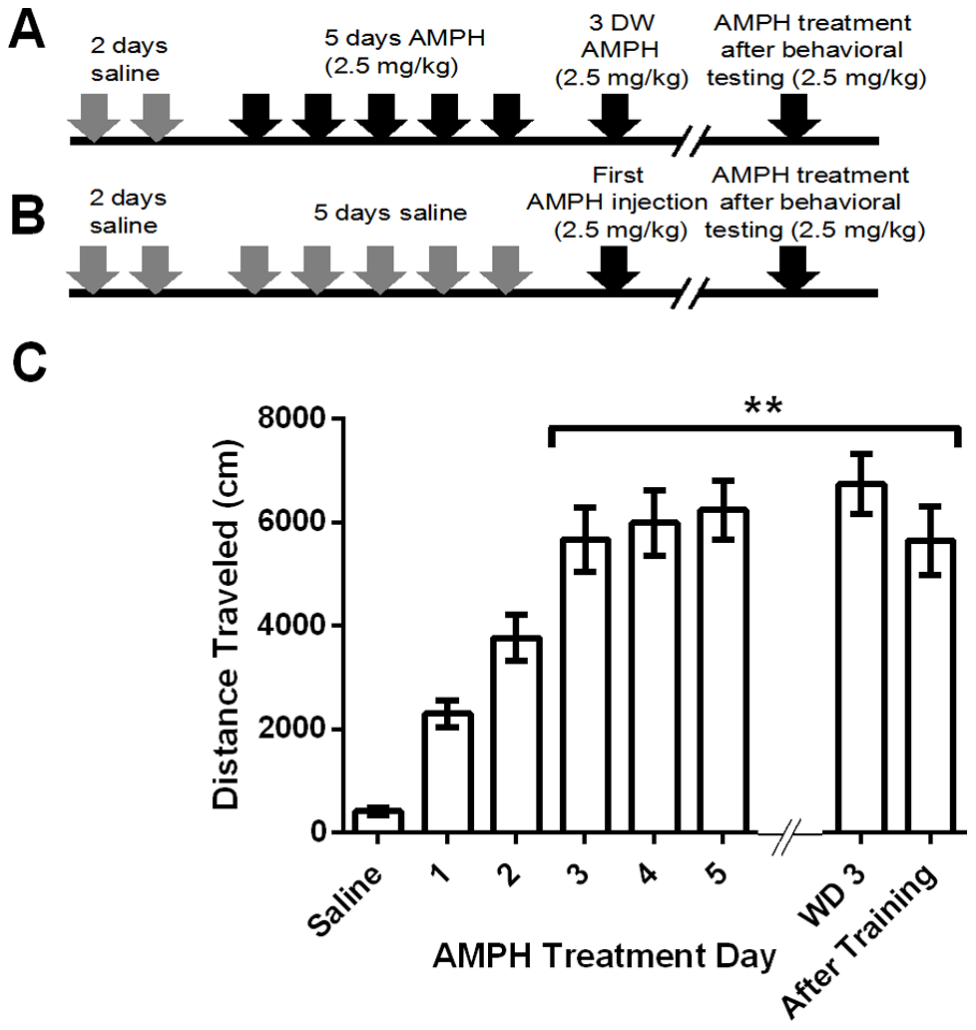
Sensitization of wild-type mice to AMPH. (A) Time course of AMPH-sensitization paradigm. (B) Time course for control (saline group). On all days, mice were injected i.p. with either saline or AMPH (2.5 mg/kg body weight) after 2-h habituation in locomotion chambers and locomotion was measured for 90 min after injection. (C) Distance traveled during 90-min period after injection of AMPH (n = 16) or saline (SAL) (n = 14) across days; one-way ANOVA of AMPH treated group: treatment effect; F(3.58, 53.76)  = 17.9; **P<0.01, 2-way, individual effect; F(15, 90) = 9.53 ***P<0.01. Tukey's multiple comparisons test shows a significant difference in locomotion between day 1 vs day 3, 4, 5, WD 3, and after training as noted by ** on graph. Data represent means ± SEM.

The sensitization protocol was performed as described [33]. C57Bl/6 mice were habituated to locomotion chambers and given injections of saline for two days, followed by injections of AMPH (2.5 mg/kg body weight, i.p.) for 5 consecutive days. After a 3-day withdrawal period (WD3) the mice were given another injection of AMPH (Fig. 1A). Control mice were given saline injections for 5 consecutive days and then given AMPH (2.5 mg/kg) for the first time 3 days later (Fig. 1B). Locomotion was measured for 90 min after each injection. There was a progressive increase in locomotion during the first 3 days of AMPH injections that then leveled off; the distance traveled in response to the third through fifth AMPH injections was about 2.5 times that of first injection (Fig. 1C; all statistics are presented in figure legends). On WD3, the AMPH-treated group continued to display and elevated locomotor response to AMPH, indicating sustained sensitization (Fig. 1C). We also challenged the sensitized mice with AMPH after all behavioral testing; the locomotor responses of the sensitized groups of mice were still significantly greater than that of the first injection (Fig. 1C). We conclude that our protocol induces robust, long-lasting sensitization that persists throughout the behavioral training period.

### Motor learning by AMPH-sensitized mice was unaffected

**Figure 2:**
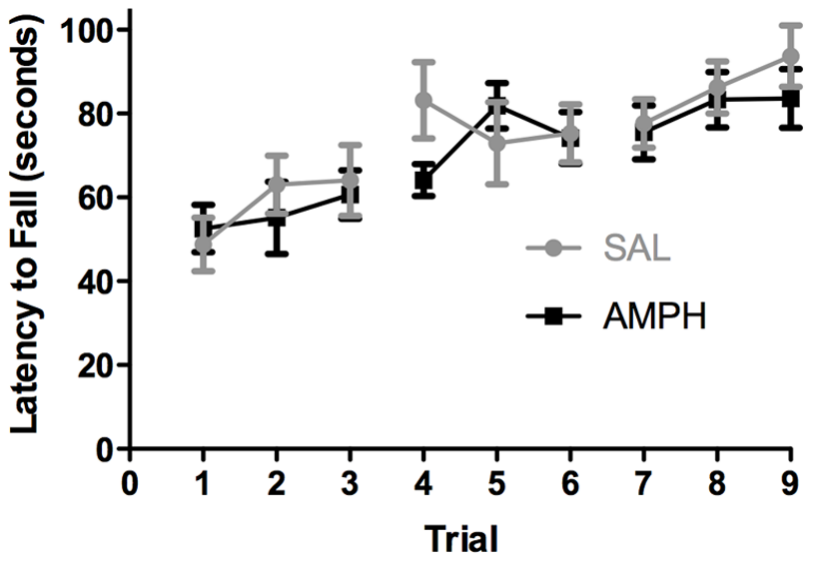
AMPH-sensitized and SAL-treated controls exhibit similar locomotor learning. Rotarod performance by wild-type AMPH (n = 14) and SAL (n = 12) control mice across days; two-way, repeated-measures ANOVA: interaction F(8,192)  = 0.99; *P* = 0.44; treatment effect F(1,24)  = 0.47; *P* = 0.50; day effect F(8,192)  = 7.76; **P<0.01. Bonferroni's multiple comparisons test showed no significant differences between the two groups in any trial. Data represent means ± SEM.

AMPH-sensitized and non-sensitized control mice were placed on an accelerating rotarod with three trials a day for three days to determine whether AMPH sensitization affects motor learning. The performance of both control and AMPH-sensitized mice improved with training, but there was no significant difference in their performance over the 9 training trials (Fig. 2). We conclude that AMPH sensitization does not affect motor learning in this task.

### Pavlovian conditioning by AMPH-sensitized mice was unaffected

**Figure 3:**
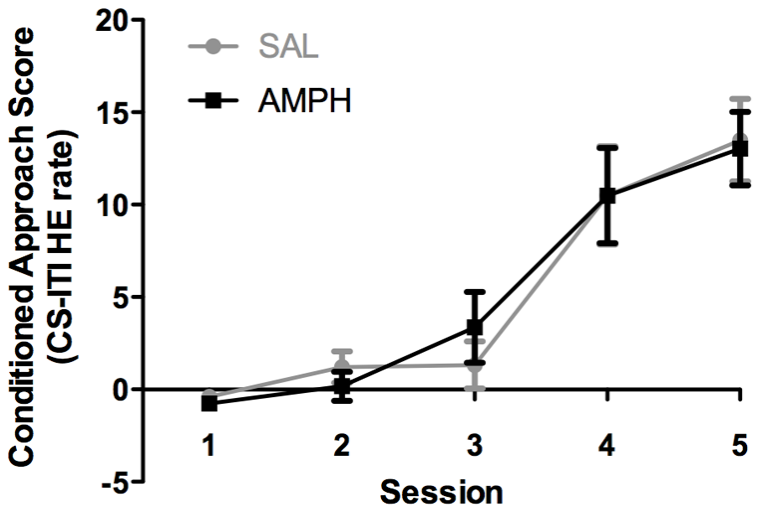
AMPH-sensitized and SAL-treated control mice exhibit similar Pavlovian conditioning. Both AMPH-sensitized (n = 8) and SAL-treated (n = 7) mice preferentially increased their head-entry (HE) rate during the conditioned stimulus (CS) relative to the inter-trial interval (ITI) to produce similar conditioned approach scores (CS-ITI head-entry rate). Two-way, repeated-measures ANOVA showed no difference between groups: interaction F(4,52)  = 0.31; *P* = 0.87; treatment effect F(1,13) <0.01; *P* = 0.98; day effect F(4, 52)  = 34.49; **P<0.01. Bonferroni's multiple comparison's test revealed no significant difference between groups on any treatment day. Data represent means ± SEM. See Fig. S1 for head-entry rates used to calculate the conditioned approach scores.

To determine whether AMPH-sensitized mice display enhanced learning of Pavlovian conditioning tasks, we tested the ability of AMPH-sensitized and control mice to learn to associate the conditioned stimulus (CS), the presentation of a lever, with the delivery of a single food pellet. Mice were given 25-min sessions on five consecutive days during which the number of head entries into the food dispenser was measured. Learning was assessed by calculating conditioned approach, which is the head-entry rate during the 10-sec CS minus the head-entry rate during the inter-trial interval (ITI). Both AMPH-sensitized and control mice learned the Pavlovian conditioning task and their conditioned approach scores increased over 10 fold during training; however, there was no significant difference in their rates of learning (Fig. 3). Plots of head-entry rates during CS and ITI for control and AMPH-sensitized groups are shown in Fig. S1. We conclude that AMPH sensitization did not affect the ability of the mice to learn that the lever cue was associated with food pellet availability.

### Water-maze learning and strategy shifting by control and AMPH-sensitized mice

**Figure 4:**
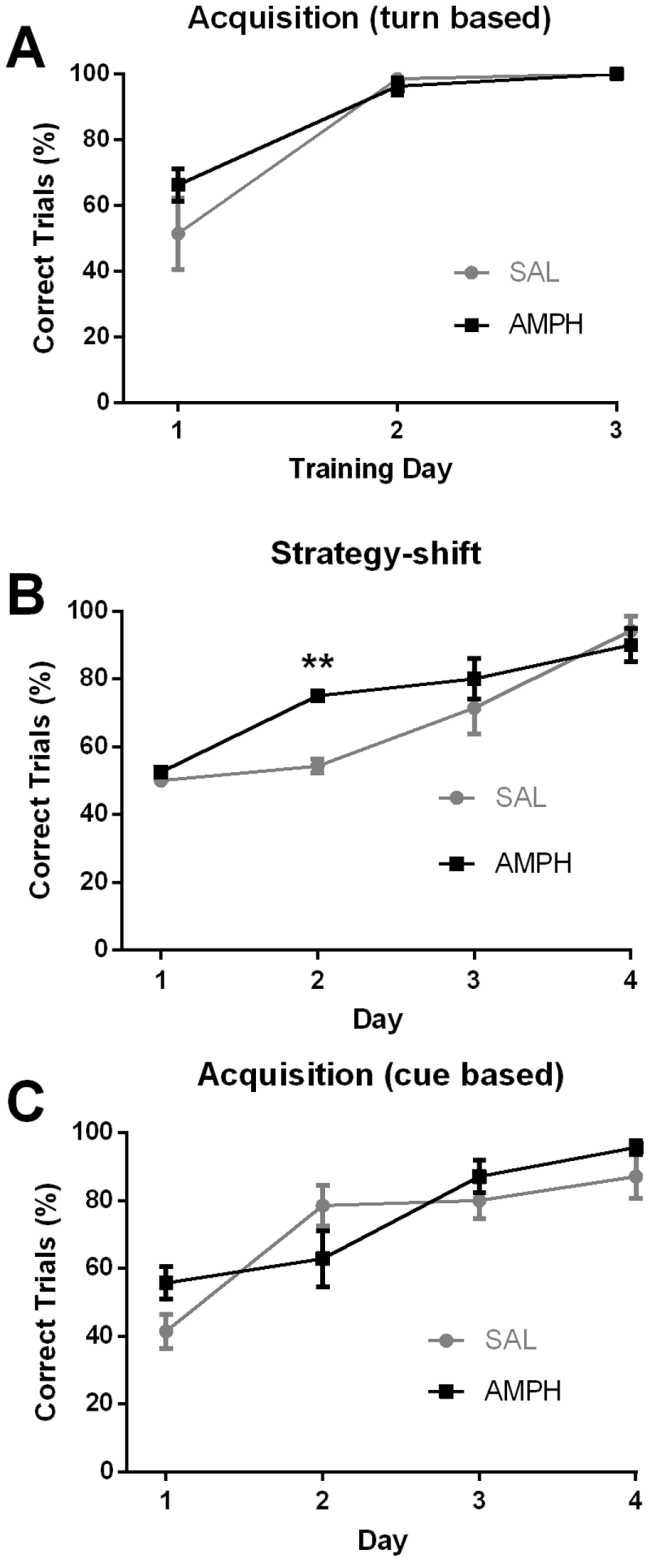
AMPH-sensitized and saline–treated control mice exhibit similar escape learning profiles. (A) Both AMPH-sensitized (n = 8) and SAL-treated control (n = 7) mice reached >95% correct trials in acquisition of turn-based learning on the second training day. There was no difference in acquisition of learning; two-way repeated-measures ANOVA: interaction F(2,26)  = 1.95; *P* = 0.16; treatment F(1,13)  = 1.03; *P* = 0.33; day effect F(2,26)  = 48.03; *P*<0.01. (B) The same groups of AMPH-sensitized and SAL-treated mice were then tested for cue-based learning. By day four, all mice reached >80% correct trials; two-way repeated-measures ANOVA including all 4 days of training: interaction F(3,39)  = 3.35; *P* = 0.03; treatment F(1,13)  = 4.01; *P* = 0.07; day effect F(3,39)  = 35.71; ***P<0.01. Bonferroni post tests showed a significant difference between treatments on day 2; **P<0.01, represented by ** on graph. (C) Acquisition of the cue-based strategy by a separate cohorts of AMPH-sensitized SAL-treated wild-type mice; both AMPH-sensitized (n = 8) and SAL-treated (n = 7) mice reached >80% correct trials on day 3. Two-way, repeated-measures ANOVA showed significant interaction and day effect showing that both groups learned, but no effect of AMPH treatment: interaction; F(3,36)  = 3.71; *P* = 0.02; treatment effect; F(1,12); *P* = 0.51; day effect; F(3,36)  = 29.8; ***P<0.01. Bonferroni's multiple comparisons test shows no difference between treatments on any day. Data represent means ± SEM.

To assess spatial learning and cognitive flexibility, we examined the ability of mice to find an escape platform using a water-based U-maze. This is essentially a T-maze with the arms bent back so that the escape platform cannot be seen from the choice point. One arm is black and the other is white. Mice were given ten trials a day over three to four days depending on the paradigm being tested. For the first test, the mice had to learn a turn-based strategy (turn left) to find the escape platform regardless of the arm color, which was changed randomly. Both AMPH- sensitized and control mice learned to make the correct choice >90% of the time by the second of three testing days with 10 trials/day. There was no difference between the two groups of mice (Fig. 4A). Subsequently, the rules changed (strategy-shift) such that the mice had to associate the color of the arm with the presence of the escape platform. Both groups learned this cue-based strategy with >90% correct choices by the fourth day after the strategy shift, but the AMPH-sensitized group was significantly better than the control group on day 2 (Fig. 4B). Most of the errors (>90%) made by both groups of mice on the first day after introducing the strategy shift were due to retention of prior turn-based strategy. To assess whether the enhanced strategy shifting seen in the AMPH-sensitized group may be due to enhanced cue-based learning, a separate cohort of AMPH- sensitized and control C57Bl/6 mice were tested first with the cue-based strategy. Both groups learned the cue-based task at the same rate (Fig. 4C). We conclude that response-based and cue-based learning in the U-maze is unaffected by AMPH sensitization, but sensitization slightly enhances shifting from one strategy to another.

### Mice lacking NMDA receptors in medium spiny neurons sensitize to AMPH

**Figure 5:**
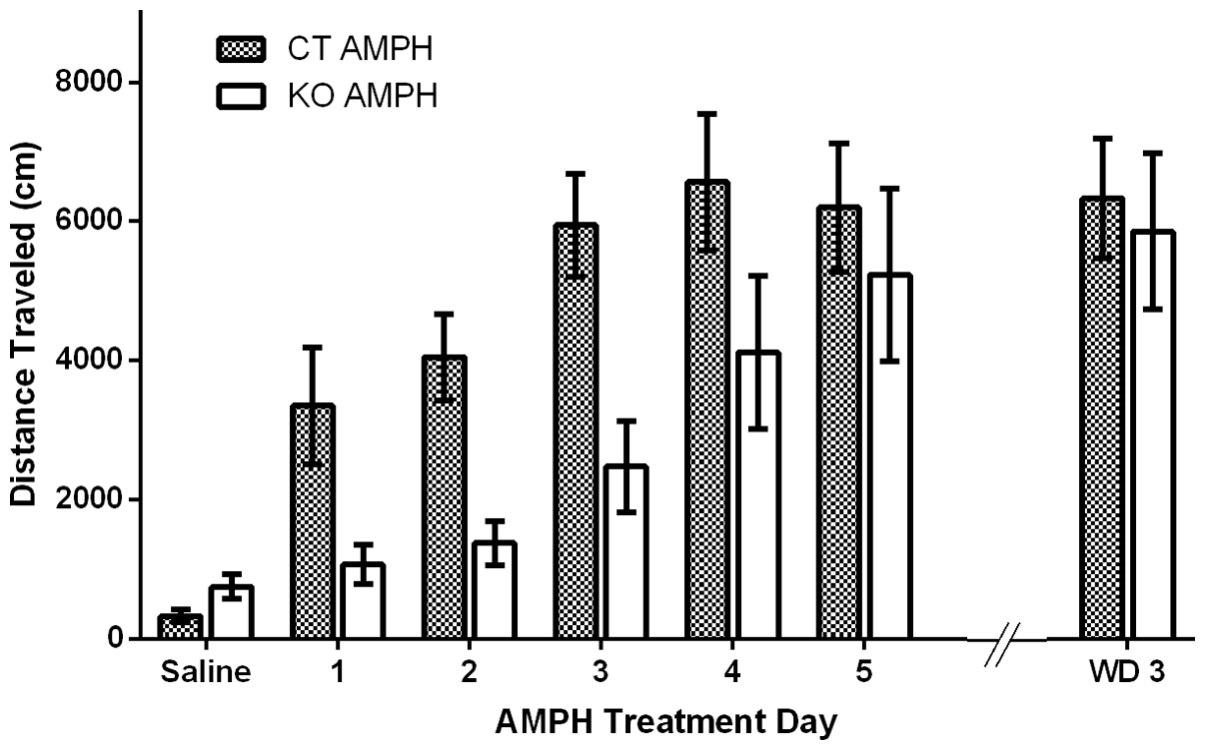
Knockout (KO) and control (CT) mice sensitize to AMPH. Mice were treated in the same paradigm as outlined in Fig. 1A. Distance traveled during a 90-min test after AMPH or SAL injection. Control SAL (n = 6); Control AMPH (n = 8 ); KO SAL (n = 8); KO AMPH (n = 8). Two-way repeated measures ANOVA: interaction; F(6,84)  = 2.62, *P = 0.022; day effect; F(5,130) = 20.46, ***P<0.01; treatment effect, F(3,26)  = 14.41, ***P<0.01. Bonferroni's post test shows a significant difference between KO and CT mice on day three, noted on the graph with *. Data represent means ± SEM.

The previous experiments revealed little effect of AMPH sensitization on learning by wild-type mice. We also asked whether AMPH sensitization would rescue the ability of mice with a learning defect due to absence of NMDA receptors in striatal MSNs to learn some of these same tasks. Mice lacking NMDA receptors in MSNs (KO mice) were generated by breeding mice with a conditional *Grin1* allele that encodes the essential NR1 subunit of NMDA receptor with mice expressing Cre recombinase from the *Gpr88* locus that encodes an orphan G-protein coupled receptor expressed in all MSNs within the striatum, as well as some other neurons [34]. These KO mice (*Grin1^Δ/lox^: Gpr88^Cre/+^*) and their controls (*Grin^lox/+^: Gpr88^cre/+^*) were heterozygous at both the *Grin1* locus and the *Gpr88* locus, but the KO mice lack NMDA receptors wherever *Gpr88* was expressed [26]. There was no difference in sensitization, rotarod or Pavlovian learning between the heterozygous control mice (*Grin^lox/+^: Gpr88^cre/+^*) and C57Bl/6 (WT) mice (Fig. S2). The KO mice and their control mice were subjected to the same sensitization protocol as before (Fig. 1A). Both KO and control mice sensitized to AMPH over the 5-day exposure and traveled the same distance on the fifth day; however, as noted previously [33], the KO mice were slower to sensitize (Fig. 5). When tested after WD3, both control and KO mice still had enhanced response to AMPH (Fig. 5). Because the NMDA receptor-deficient mice were sensitized to AMPH by repeated exposure to the drug, it was reasonable to ask whether the changes that occurred during sensitization would rescue learning in any of the paradigms that were examined in wild-type mice.

### Prior AMPH sensitization did not affect motor learning by control or KO mice

**Figure 6:**
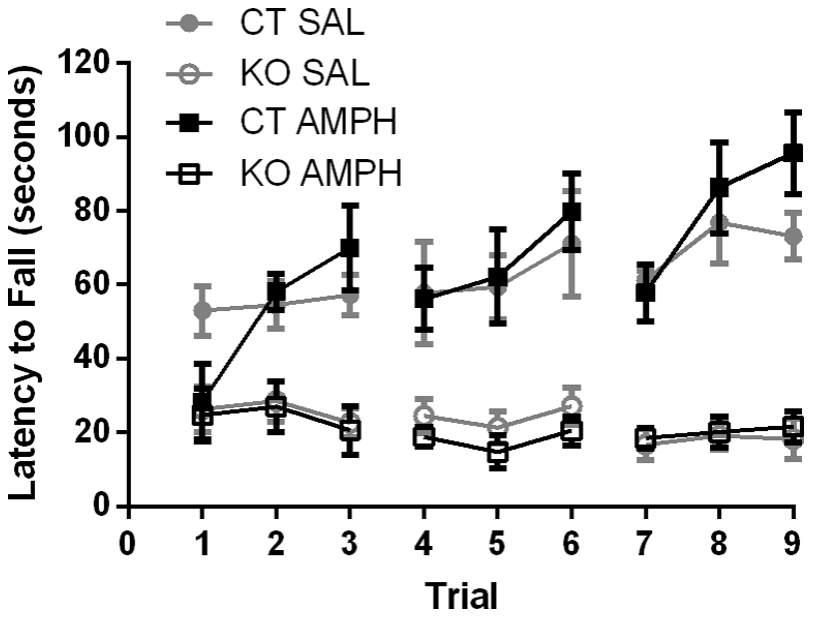
Sensitized knockout mice do not show increased locomotor learning. Rotarod performance by control AMPH-sensitized (n = 6) and SAL-treated mice (n = 5) was the same and both groups demonstrated motor learning over time. Rotarod performance by knockout AMPH-sensitized (n = 8) and SAL-treated (n = 8) mice were equivalent; knockout AMPH-treated mice showed no improvement with training. Two-way, repeated-measures ANOVA: interaction F(24, 176)  = 3.13; *P*<0.01; treatment F(3,22)  = 29.21; *P*<0.01; day effect F(24,176)  = 3.133; *P*<0.01. Tukey's multiple comparisons test shows a significant difference between the performance of control (CT) and KO mice, but no significant difference between the performance of SAL vs. AMPH treatment in either the KO or CT groups. Data represent means ± SEM.

To examine motor learning, AMPH-sensitized KO and control mice were tested on an accelerating rotarod. The control mice improved their performance over the 9 trials, but prior AMPH sensitization had no effect on motor learning (Fig. 6), in agreement with our previous results with wild-type mice (Fig. 2). The KO mice performed poorly on the rotarod task and did not improve with training as described [26]. Prior AMPH sensitization of the KO mice did not improve their rotarod performance compared to the non-sensitized KO animals (Fig. 6). Thus, sensitization, which enhanced the locomotor response of KO mice to AMPH, did not improve their ability to stay on the rotating rod.

### Prior AMPH sensitization did not affect performance of the Pavlovian-conditioning task by control or KO mice

**Figure 7:**
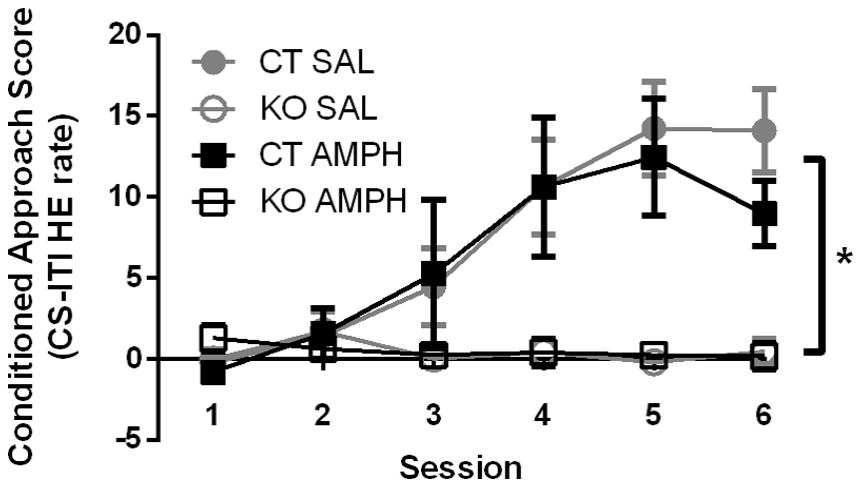
AMPH-sensitized knockout mice do not manifest Pavlovian conditioning. Control SAL-treated (n = 6) and AMPH-sensitized (n = 8), mice increased their head-entry (HE) rate to cued stimulus (CS) relative to inter trial interval (ITI) during learning resulting in a significant conditioned approach scores for both groups. Both knockout AMPH-sensitized (n = 7) and SAL-treated (n = 8) mice failed to learn the paradigm. Two-way, repeated-measures ANOVA: interaction F(15,125)  = 4.41; *P*<0.01; treatment effect F(3,25)  = 7.86; *P<*0.01; day effect F(5,125)  = 10.28; ***P<0.01. Tukey's multiple comparisons test showed a significant difference between performance of KO and CT mice in both treatments, but no significant difference in performance between the sensitized and non-sensitized groups of either genotype, represented by * on graph. Data are means ± SEM.

We also tested the KO mice and their controls in the Pavlovian conditioning paradigm. In agreement with the results obtained with wild-type mice, AMPH sensitization did not affect learning the association of the CS with the US (food reward) by the control mice (Fig. 7). The KO mice were unable to learn the association and hence showed no change in conditioned approach during the 5 training days in agreement with previous results [26]. KO mice have normal baseline and spontaneous locomotion, and display normal exploratory behavior [26]. AMPH sensitization of the KO mice did not improve their ability to perform in this Pavlovian task (Fig. 7). Examination of head-entry rates during CS presentation and the ITI revealed that on day 1 of training, the KO and control mice had similar head-entry rates during CS presentation and ITI and both groups ate most of the pellets dispensed, suggesting that they were equally curious about the food dispenser and had similar preferences for the pellets (Fig. S3).

## Discussion

Our experiments reveal that prior AMPH sensitization has little effect on learning new tasks including the ability to stay on an accelerating rod, making an association of a cue with reward availability, or learning a water-based U-maze using either a turn-based or cue-based strategy. Importantly, learning all of these behaviors depends on dopamine and glutamatergic (via NMDA receptors) signaling within the striatum [24], [26], [35]. We originally chose AMPH for these studies because it releases dopamine into the extracellular space independently of dopamine neuron activity; however, recent data indicate that AMPH potentiates ongoing phasic dopamine signaling *in vivo* [36]. The sensitization protocol that we used involves treating mice for 5 consecutive days in a novel environment with another dose after 3 days of withdrawal. This procedure results in robust locomotor sensitization; we tested them again after performing all behavioral tests and showed that they were still sensitized. This protocol produces behavioral sensitization even in mice that lack NMDA receptors in either their dopamine neurons or all MSNs [27], [33]. GPR88 is expressed in all MSNs, but also in a few other neurons within the brain [34]; thus, we cannot rule out the contribution of NMDA receptor signaling in non-MSNs to the behavioral deficits of the KO mice. Because of the robust behavioral sensitization, we assume that changes in synaptic plasticity, signaling, and transcription that have been well described by others also occurred in this study. Furthermore, the differences observed between the sensitized and non-sensitized animals is due to sensitization to AMPH rather than just exposure to AMPH, as all non-sensitized animals in the study received one injection of AMPH before participation in learning paradigms.

We anticipated the synaptic changes that allow sensitization to occur in the absence of NMDA receptors in MSNs might facilitate learning by those mice. However, we observed no learning improvement by the KO mice in any of the tasks examined. Thus, whatever events allowed behavioral sensitization did not enhance natural learning processes. Even in C57Bl/6 mice, the only learning that was affected by prior AMPH sensitization was slightly improved performance in a strategy-shift experiment. A similar study with rats revealed no effect of AMPH sensitization on their performance of a strategy-shift experiment or many other paradigms including working-memory tests; however, there was a deficit in one aspect of an attention set-shifting experiment [37]. Harmer and Phillips [38] showed that AMPH sensitization of rats enhanced their acquisition of a conditioned response to a cue that predicted food reward, contrary to what we observed, and Roesch et al [39] showed that sensitized rats were more sensitive to reward size and delivery time. Different conditioning cues, extents of sensitization and/or species might account for the lack of effect in our study.

Previous experiments demonstrate that psychoactive drug treatments alter synaptic properties in VTA dopamine neurons. Changes in the AMPA to NMDA receptor ratio were observed in dopamine neurons 24 h after a single injection and persisted for a few days [10]. After multiple injections, such as that used in our sensitization protocol, the synaptic changes persisted for weeks [31]. In dopamine neurons, the increase in AMPA/NMDA receptor ratio reflects an increase in the number of AMPA receptors transported into the synapse, a change in the subunit composition of those AMPA receptors, and a reduction in NMDA receptor function. These changes in glutamate signaling are accompanied by a decrease in GABAergic signaling onto dopamine neurons [18]. The net effect is that dopamine neurons become more excitable, which probably underlies the enhanced dopamine release in the striatum that occurs after sensitization with either cocaine or AMPH [21], [40]. These drug-induced changes in receptors resemble the LTP that occurs in other brain regions during learning [18]; importantly, the changes in receptors that occur *in vivo* preclude the ability to induce additional LTP *in vitro*. The AMPA to NMDA receptor ratio increases in dopamine neurons as animals learn a Pavlovian association, suggesting that the increase promotes reward learning [31]–[32], although LTP-like changes can occur in dopamine neurons without being rewarding [41].

In the NAc, treatment with psychostimulants lowers the intrinsic excitability of MSNs and their AMPA to NMDA receptor ratio [16]. This process of LTD is observed when measured in striatal slices shortly after drug treatment, and protocols that normally induce LTD are no longer effective. However, after a period of withdrawal (when our behavioral tests were performed), the situation reverses such that there is an increase in the AMPA/NMDA receptor ratio, an accumulation of GluR2A-lacking AMPA receptors, and re-establishment of LTP in D1 receptor-bearing MSNs [3], [18]. These changes in synaptic plasticity are accompanied by many other adaptations including changes in extracellular glutamate, intracellular signaling, transcription and chromatin structure [2]–[6], [16]. The increase in AMPA to NMDA receptor ratio in MSNs could reflect LTP or it might represent a form of homeostatic plasticity caused by the reduced glutamatergic signaling in the striatum [16], [42]. Importantly, behavior sensitization can be reversed by optogenetic techniques that reverse the LTP in D1-receptor-bearing MSNs; thus, behavioral sensitization appears to be directly correlated with maximal LTP in the direct-pathway MSNs [3]. The adaptations that transpire in MSNs and dopamine neurons in response to sensitization are thought to underlie addiction-like behaviors. A large body of research has documented that psychoactive drugs promote learning about cues that predict drug-induced effects [20], [43]–[45]. Sensitized rats acquire drug self-administration more readily [46], they will work harder to obtain drugs [47]–[48], and they escalate their drug intake more rapidly [49]. The inability to generate LTD in striatal slices is also correlated with addictive behavioral phenotypes in rats [50]. Sensitization also increases the incentive value of non-drug rewards such as sugar, food or access of males to a receptive female as well as cues that predict such rewards [51]–[53], suggesting that sensitization causes excessive cue-triggered incentive salience (wanting) for an associated reward [52]. Thus, despite the lack of effect of AMPH sensitization in learning paradigms that we examined, it may affect learning of other tasks.

Does sensitization to drugs of abuse induce the same synaptic changes that normally underlie dopamine-dependent learning? If the changes in LTP have already occurred either in dopamine neurons or their MSN targets, then how can an animal learn anything new after sensitization? To illustrate the problem consider an appetitive Pavlovian learning task in which presentation of a cue predicts the availability of a food reward. Early in the learning process, the animal does not pay much attention to the food dispenser but with training the animal gradually learns to approach the food dispenser primarily when the cue is presented. This shift in attention is reflected as changes in dopamine neuron activity [54]–[55] and dopamine release [32], [56]–[57]. At first, dopamine is released in the NAc only to the unexpected food reward but with training dopamine is released in response to the cue. Dopamine signaling in the NAc is essential for Pavlovian learning, especially acting on D1 receptor-bearing MSNs [57]–[58] and it is associated with changes in activity of MSNs in the NAc [59]. Our results showing no effect of AMPH sensitization on learning a Pavlovian association are consistent with previous results showing that cocaine sensitization does not influence learning a Pavlovian association by rats [60], although they contrast with the results of Harmer and Phillips [38]. Genetic experiments reveal that Pavlovian learning depends on the presence of NMDA receptors in the prefrontal cortex and the MSNs, but not in dopamine neurons themselves [61]. Because NMDA receptor signaling is necessary in most neurons, including MSNs, to establish LTP [25], [30], [33], [62]–[63], LTP appears to be necessary in the prefrontal cortex and striatum for Pavlovian conditioning [61].

Even though mice lacking NMDA receptors in dopamine neurons have reduced ability to burst fire [29], [64], release less dopamine *in vivo* [61], and do not undergo LTP [27]–[28], they can learn a Pavlovian association normally [61] as well as most other tasks, albeit more slowly than control mice [64]. However, pharmacological infusion of NMDA receptor antagonists into the VTA prevents sensitization [65], [66], [67] and learning a conditioned place preference or a Pavlovian association [32], [68]. These antagonist effects may be mediated by non-dopaminergic neurons in the VTA [68] or perhaps they are the consequence of off-target actions of the antagonist. Despite the loss of NMDA receptors from dopamine neurons, the AMPA receptor current is elevated, perhaps as compensation, and cannot be increased further by psychostimulant administration [27]. Thus, an alternative explanation for not requiring NMDA receptors in dopamine neurons for learning is that LTP has already happened. If that explanation is correct, then the consequences of removing NMDA receptors from cortical or striatal neurons are fundamentally different than their loss from dopamine neurons because their loss prevents learning in the former case but not the latter.

From the discussion above we conclude that NMDA receptor signaling is more important in MSNs (especially the D1 receptor-bearing MSNs) than in dopamine neurons for learning new tasks but they are dispensable in both brain regions for AMPH sensitization. Hence, NMDA-dependent LTP in all MSNs does not appear to be necessary for sensitization, but LTP or some other consequence of NMDA receptor currents in MSNs is required for learning the tasks studied here. Sensitization can also occur independently of changes in AMPA receptors in the NAc [69]. Our behavioral experiments indicate that the changes that accompany sensitization to AMPH have little or no effect on normal learning processes that depend on dopamine signaling in the striatum despite the observation that some of these changes appear to affect most dopamine or MSNs that are sampled. The nearly uniform change in synaptic plasticity measured either in dopamine neurons or MSNs in response to psychostimulants [3], [10]–[18], [31] are surprising considering that the responses of NAc neurons to cocaine and natural rewards (food or water) are largely segregated even in rats that have been self-administering cocaine for an extended time [70]. Likewise, only a subset (2–3%) of neurons is activated in the NAc in response to a specific environment associated with cocaine sensitization, and importantly, inactivation of those neurons eliminates the context-specific responses to cocaine without affecting other locomotor responses [71]. The latter result indicates that the function of a small subset of NAc neurons is necessary for the manifestation of the association between cocaine and a specific place.

A resolution to the conundrum that sensitization has little effect on learning about natural rewards is that the critical synaptic changes that occur during learning take place outside of the dopamine and striatal neurons themselves. The altered properties of dopamine and striatal neurons may enhance the incentive salience of cues and performance of tasks related to the drug experience, but the synaptic plasticity that underlies learning occurs in brain regions that are less affected by drugs that elevate extracellular dopamine. Synaptic plasticity in neurons of amygdala, hippocampus, cortex, and/or thalamus that send their glutamatergic projections to the striatum could be responsible for learning while the adaptations in the dopamine neurons and MSNs that occur with sensitization may reflect homeostatic changes to altered dopamine signaling. The convergence of glutamatergic and dopamine signals on MSNs could be necessary to pay attention to relevant environmental stimuli and disruption of either signal may compromise paying attention to salient events and hence the ability to learn to make associations and initiate appropriate actions. The synaptic, signaling and morphological changes that occur in the striatum in response to repeated drug exposure may represent homeostatic adaptations that facilitate normal learning despite changes in dopaminergic and glutamatergic inputs to striatal MSNs.

## Methods

### Mice

All mouse lines used in these experiments were backcrossed to C57Bl/6 mice for >10 generations. The wild-type mice were C57Bl/6. Two cohorts of sensitized C57Bl/6 were tested. Both participated in rotarod training and Pavlovian conditioning. One cohort was tested in the water U-maze with a turn-based acquisition followed by a strategy-shift, and the other cohort was tested only for cue-based acquisition. The sequence of behavioral tests was consistent for all groups. The KO mice were *Gpr88^Cre/+^: Grin1^lox/Δ^* and their controls were *Gpr88^Cre^* ^/*+*^: *Grin1^lox/+^* [33]; hence, both groups were heterozygous for *Gpr88* and *Grin1* in all cells except the striatal MSNs of KO mice, which were homozygous for loss of *Grin1*. Two cohorts of sensitized KO and control mice were sensitized and then tested in rotarod and Pavlovian conditioning paradigms as above. The sequence of the two behavioral tests was reversed for each group. Approximately equal numbers of male and female mice were used in all experiments; because no differences between males and females were observed in any of the behaviors tested, data from both sexes were combined. All animals were between 7 and 9 weeks of age at the start of experiments. All animal protocols were performed in accord with NIH guidelines and approved by the University of Washington Institutional Animal Care and Use Committee (protocol 2183-02).

### AMPH sensitization

On days 1–2, animals were habituated to activity chambers (Columbus Instruments) for 2 h before receiving saline (10 µl/g, i.p.), after which locomotion was monitored for an additional 90 min. On days 3–7, and day 11 [3-days of withdrawal (WD3)], animals were placed in locomotion chambers for 2 h before receiving AMPH (2.5 mg/kg; Sigma, 10 µl/g, i.p.), and their locomotion was monitored for an additional 90 min. The same protocol was used to measure AMPH sensitization in all experiments. Behavior testing began 5 to 7 days after WD3 AMPH injection. After training, one group of AMPH-sensitized and control mice was given a challenge dose of AMPH at WD22; a second group was challenged on WD46. The difference in withdrawal testing days was due to differences in final training days between the two groups. Non-sensitized animals received saline on days 1–7. On days 3 and 22 or 46 (depending on which test group they were in) the non-sensitized animals also received AMPH (2.5 mg/kg; Sigma, 10 µl/g, i.p.).

### Rotarod

Mice were placed on an accelerating rod (Rotamex 4/8; Columbus Instruments) that increased in speed from 5 to 55 rpm over the course of a 5-min trial. Animals were given three trials a day, separated by 15 min, for three days. Latency to fall is reported for each trial.

### Pavlovian conditioning

All training was performed in operant conditioning chambers (ENV-307W; Med Associates). Mice were given 5 days of conditioning, which consisted of 25 CS and 25 US pairings in which a 10-s lever presentation was immediately followed by the delivery of a food reward. Learning was measured as the increase in CS-elicited head-entry rate (CS–HE rate) to the food receptacle relative to inter-trial interval head-entry rate (ITI–HE rate), and reported as a conditioned approach (CA) score calculated as the difference between CS–HE and ITI–HE rates for each mouse. See reference [61] for a more detailed explanation of the protocol.

### Water-based U-maze

A water-based U-maze with two arms bending back towards the stem, one painted white and the other black was used. The maze was constructed out of galvanized metal. Mice were released in one end of the stem, with the junction to the two arms at the other end. Due to the curve of the arms the mouse was not able to see the platform from the decision point. The right-left orientation of the black and white arms was altered randomly between trials so that both colors were located on both sides equally throughout the session. Mice were given 10 trials a day over three to four days depending on the paradigm tested. The first turn the mouse made (body entirely in one of the arms) was scored as correct if it chose the arm with the platform or incorrect if it chose the arm without the platform, and then the mouse was left in the maze until the platform was found. For more detailed methods see [24].

## Supporting Information

Figure S1 **Wild-type AMPH-sensitized and SAL-treated control mice manifest similar Pavlovian conditioning.** (A) SAL mice (n = 14) increased their CS-HE rate relative to their baseline ITI-HE rate during training; two-way repeated measures ANOVA; interaction F(4,48) = 5.67; ***P<0.001; day effect F(4,48) = 25.23; ***P<0.001; HE rate effect F(1,12) = 16.17; **P = 0.002. Bonferroni's multiple comparisons test shows significant difference between HE rates on day 4 and 5, represented by ** on graph. (B) AMPH-sensitized mice (n = 16) increased their CS-HE rate relative to their baseline ITI-HE rate during training; two-way repeated-measures ANOVA: interaction F(4,56)  = 10.56; ***P<0.001; day effect F(4,56)  = 34.91; ***P<0.001; HE rate effect F(1,14)  = 7.78; *P = 0.015. Bonferroni's multiple comparisons test shows significant difference between HE rates on day 4 and 5, represented by *** on graph. Data are means ± SEM.
(TIF)

Figure S2 **Heterozygous and wild-type mice behave similarly during sensitization, motor learning, and Pavlovian conditioning.** (A) Heterozygous (HET) *Grin^lox/+^: Gpr88^cre/+^* (n = 8) and wild-type (WT) *Grin^+/+^: Gpr88^+/+^* C57Bl/6 mice (n = 16) sensitize similarly to AMPH. Two-way repeated-measures ANOVA; interaction F(6, 132)  = 0.54; *P* = 0.77; day effect F(6,132)  = 50.75; *P*<0.01; treatment effect F(1,22)  = 0.13; *P* = 0.73. Bonferroni's multiple comparisons test showed no significant difference between CT and WT sensitization. (B) Rotarod performance of non-sensitized HET (n = 5) and WT mice (n = 12) were comparable; two-way, repeated-measures ANOVA; interaction F(8,120)  = 0.67; *P*  = 0.72; day effect F(8,120)  = 3.52; *P*<0.01; treatment effect F(1,15)  = 1.50; *P*  = 0.24. Bonferroni's multiple comparisons test showed no significant difference between CT and WT performance in any trial. (C) Both non-sensitized HET (n = 6) and WT mice (n = 7) mice preferentially increased their head-entry (HE) rate during the conditioned stimulus (CS) relative to the inter-trial interval (ITI) to produce similar conditioned-approach scores (CS-ITI head-entry rate). Two-way repeated-measures ANOVA; interaction F(4,44)  = 0.26; *P* = 0.90; day effect F(4,44)  = 24.28; *P*<0.01; treatment effect F(1,11)  = 0.30; *P* = 0.60. Bonferroni's multiple comparisons test showed no significant difference between CT and WT performance on any of the days tested.
(TIF)

Figure S3 **Neither AMPH-sensitized nor SAL-treated knockout mice learned Pavlovian conditioning, controls learned the same regardless of AMPH-sensitization.** (A) Control SAL-treated mice (n = 6 ) increased their CS-HE rate relative to their baseline ITI-HE rate during training. Two-way repeated measures ANOVA: interaction F(4,40)  = 7.322 ***P <0.001; day effect F(4,40)  =  21.44; ***P ¸0.001; HE Rate effect F(1.10)  = 4.65; *P* = 0.057. Bonferroni's multiple comparisons test shows significant difference between HE rates on day 4 and 5, represented by * on graph.(B) Knockout SAL-treated mice (n = 8) did not increase their CS-HE rate relative to their baseline ITI-HE rate during training; two-way repeated measures ANOVA: interaction F(4, 56)  = 3.017; *P* = 0.347; day effect F(4.56) = 3.02; *P = 0.025; HE rate effect; F(1,14)  = 0.197; *P* = 0.663. (C) Control AMPH-sensitized mice (n  =  8) increased their CS-HE rate relative to their baseline ITI-HE rate during training; two-way repeated-measures ANOVA: interaction F(4,56)  = 2.38; *P* = 0.06; day effect F(4,56)  = 8.16; *** *P*<0.001; HE rate effect F(1.14)  = 3.09; *P* = 0.10. Bonferroni's multiple comparisons test shows significant difference between HE rates on day 5, represented by * on graph. (D) Knockout AMPH-sensitized mice (n = 7) did not increase their CS-HE rate relative to their baseline ITI-HE rate during training; interaction F(4,48)  = 0.245; *P* = 0.91; day effect F(4.48)  = 0.698; *P* = 0.597; HE rate effect F(1.12)  = 0.37; *P* = 0.55.Data represent means ± SEM.
(TIF)
